# New Appliances and Things Medical

**Published:** 1893-05-27

**Authors:** 


### NEW APPLIANCES AND THINGS MEDICAL.

[All preparations, appliances, novelties, etc., of which a notice is desired, should be sent for the Editor, to care of The Manager, 428, Strand, London, W.C.]

### DIGESTIVE AGENTS AND CONCENTRATED FOOD STUFFS.

(ARMOUR AND CO., CHICAGO, ILLINOIS, ; 59 AND 60, TOOLEY STREET, LONDON, S.E.)

We have received a number of Armour's digestive agents and concentrated food preparations for investigation and report. In entering upon this investigation, we naturally expected a very high standard of excellence, because the firm is well-known as one of the largest manufacturers and exporters of the best forms of canned meat goods. Taking the digestive agents first. The two chief factors in judging these are (*a*) pleasant flavour in themselves and their products; (*b*) power of reducing the insoluble albuminous food to the soluble peptone condition. The specimens we received consisted of (*a*) tablets (three grains) of pure pepsin, (*b*) essence of pepsin, (*c*) tablets of pancreatin and soda for predigesting foods, especially milk. (*a*) On trial with the usual hard boiled white of egg, we found them to quickly reduce the masses of coagulated albumen to the soluble form. The solution is not offensive to taste or smell. (*b*) This is a remarkably pleasant tasting solution of pepsin quickly reducing albumen to the peptone condition. It is much more palatable than any other fluid preparation of pepsin with which we are acquainted. (*c*) These tablets are most active in their peptonising powers, and their solution is not offensive to taste or smell. The idea of combining the pancreatin with the alkaline salt is a good one. Each tablet is for half-a-pint of milk. We gave a portion of the sample to a nurse who was attending a case for us. She expressed her pleasure not only at the increased facility in using the material, but also at the very complete peptonisation of the milk. It will be seen that these preparations all carry out the two conditions of activity of digestion and absence of disagreeable taste or odour. Armour's Nutrient Wine of Beef Peptone: On examination we found abundant evidence of the presence of peptones. It contains in every pint the peptone resulting from the digestion of one pound of the best lean beef, quite free from fat. The question to answer with regard to this preparation from the physician's point of view is in what way is this better than the other beef wine preparations? The answer is that they are merely stimulants added to a stimulant, viz., extract of meat added to wine, whilst Armour's makes soluble albumen added to wine, that is, nutriment plus stimulation. It is a very pleasant and excellent form of medicament worthy of general use. We also received a pot of Armour's Extract of Meat. We find this to be well made, well flavoured, and with excellent keeping properties. In conclusion, we may state why we should expect Armour's digestive preparations should have a high standard of excellence: (*a*) Because the firm is one of the largest in the world; (*b*) because it has the pick of highly-bred animals in the chief cattle market of the world—Chicago. These two factors of large business and immense area of selection enable Armour's to manufacture in the best way from the best material, and the result is, as we have stated, a series of preparations of the highest activity and value combined with a really pleasant flavour.

